# Opioid overdose counseling and prescribing of naloxone in rural community pharmacies: A pilot study

**DOI:** 10.1016/j.rcsop.2021.100019

**Published:** 2021-05-03

**Authors:** Benjamin S. Teeter, Mary M. Thannisch, Bradley C. Martin, Nickolas D. Zaller, Duane Jones, Cynthia L. Mosley, Geoffrey M. Curran

**Affiliations:** a Center for Implementation Research, Department of Pharmacy Practice, University of Arkansas for Medical Sciences, Little Rock, AR 72205, United States of America; b University of Arkansas for Medical Sciences, Little Rock, AR 72205, United States of America; c Division of Pharmaceutical Evaluation and Policy, Department of Pharmacy Practice, University of Arkansas for Medical Sciences, Little Rock, AR 72205, United States of America; d Fay W. Boozman College of Public Health, University of Arkansas for Medical Sciences, Little Rock, AR 72205, United States of America; e Harps Food Stores, Inc., Springdale, AR 72762, United States of America; f Center for Implementation Research, Departments of Pharmacy Practice and Psychiatry, University of Arkansas for Medical Sciences, Little Rock, AR 72205, United States of America; g Central Arkansas Veterans Healthcare System, 2200 Fort Roots Drive, North Little Rock, AR 72114, United States of America

## Abstract

**Introduction:**

Fatal overdoses from opioids increased four-fold from 1999 to 2009, and they are now the leading cause of death among Americans under 50. Legislation has been passed by every state to increase access to naloxone but dispensing by community pharmacies remains low.

**Objectives:**

The objective of this study was to pilot test a proactive opioid overdose counseling intervention and a passive naloxone intervention, and the implementation strategies developed to support their delivery, in rural community pharmacies on relevant implementation outcomes.

**Methods:**

The interventions, implementation strategies, and the overall pilot study approach were developed in a collaborative partnership with a regional supermarket pharmacy chain. They selected 2 rural pharmacies to participate in the pilot study and 2 non-intervention pharmacies to serve as comparison sites. Two interventions were pilot tested in the 2 intervention pharmacies: 1) a proactive opioid overdose counseling intervention and 2) a passive naloxone intervention. An explanatory sequential mixed-methods design was utilized to evaluate adoption, feasibility, acceptability, and appropriateness outcomes after the 3-month observation period.

**Results:**

Between the 2 intervention pharmacies, 130 patients received the opioid overdose counseling intervention. 44 (33.8%) were prescribed and dispensed naloxone. Zero naloxone prescriptions were written or dispensed at the comparison pharmacies. Interviews with pharmacy staff found the interventions to be feasible, acceptable, and appropriate in their settings.

**Conclusion:**

This small scale pilot study in partnership with a regional supermarket pharmacy chain had positive results with a third of patients who received the opioid overdose counseling intervention being dispensed naloxone. However, the majority of patients did not receive naloxone indicating additional revisions to the intervention components and/or implementation strategies are needed to improve the overall impact of the interventions.

## Introduction

Opioids are among the most effective drugs for acute and severe pain and are widely considered appropriate for pain associated with cancer, most surgeries, and traumatic injuries.^1^, ^2^, ^3^ The use of opioids for chronic non-cancer pain (CNCP) has increased dramatically, paralleled by increased rates of opioid abuse, dependence, and overdose deaths.^4^ The number of opioid prescriptions in the US reached 259 million in 2012, nearly a fourfold increase since 1999.^5^ The problem is particularly acute in the southern region of the US where 6 of the 7 states with the highest opioid prescribing rates are located.^6^ From 1999 to 2012 a total of 165,000 Americans died of opioid overdose, leading the Centers for Disease Control and Prevention (CDC) to label opioid abuse and overdose an “epidemic.” In the 12-month period ending June 2020, more than 83,000 drug overdose deaths occurred in the US – an increase of over 21% compared to the previous year and the highest number of overdose deaths ever recorded in a 12-month period.^7^ While much of this increase in overdose deaths can be attributed to rise in use of illicit fentanyl, a third of overdose deaths involve at least 1 prescription opioid.^8^ It is estimated that opioids could kill nearly 500,000 Americans in the next decade.^9^

Inappropriate use of opioid medications can lead to a wide range of clinical events including violence, accidents, and overdose.^9^^,^^10^ Dosages above 50 morphine milligram equivalents (MME) per day increase risk for overdose by at least 2 times the risk of dosages less than 20 MME per day.^11^ For dosages at or above 90 MME per day, the risk for overdose increases by greater than 10 times the risk at less than 20 MME per day.^11^ Additionally, concurrent use of opioids and benzodiazepines, muscle relaxers, or hypnotics increases overdose risk and is discouraged in the CDC guidelines for opioid prescribing.^12^ Other risk factors include longer duration of treatment,^13^ multiple providers,^14^ history of non-opioid substance use disorder,^15^ history of mental health disorder,^16^ and underlying respiratory conditions.^17^ Additionally, overdoses have been seen in household members of people in possession of prescription opioids.^18^

Naloxone hydrochloride (naloxone) is an opioid antagonist that reverses the potentially fatal respiratory depression caused by opioids.^19^ Naloxone was approved for the treatment of opioid overdose by the FDA in 1971 and has since been available from emergency medical service (EMS) providers, first responders, and emergency department clinicians. Over the past 10 years, every state has enacted legislation to increase naloxone access.^20^ In Arkansas, where this study was conducted, a statewide standing order pursuant to Act 284 of 2017 (SB 142) (Arkansas Code § 17–92- 101(16)) authorized licensed pharmacists to order, dispense and/or administer naloxone. Other states have enacted laws that allow pharmacists to prescribe and dispense naloxone through a collaborative practice agreement with a physician or dispense naloxone without a prescription. However, despite these efforts to increase access to naloxone, analysis of retail pharmacy data from 2012 to 2018 showed low implementation of prescribing and dispensing of naloxone by pharmacies.^21^

Previous research conducted in over 400 large chain pharmacies in Massachusetts and Rhode Island tested an intervention that was designed to promote awareness and encourage patient-initiated conversations with pharmacists about naloxone.^22^ The intervention included an online continuing education course for pharmacists, a collaborative practice agreement with a physician to provide naloxone, and a naloxone education pamphlet that was to be used to counsel patients receiving naloxone in the pharmacy. Additionally, various forms of advertisement, like posters, were created to promote awareness of naloxone. The overall intervention approach was passive, meaning the patient/customer was required to initiate the conversation with the pharmacist about naloxone. The results from this study were positive, increasing interest in obtaining naloxone and naloxone dispensed to a population that was predominantly injectable drug users. Southern, predominantly rural states have the highest opioid dispensing rates in the U.S. and a large number of small, rural pharmacies that have different needs and workflows when compared to large, urban, chain pharmacies.^6^ This study pilot tested a proactive opioid overdose counseling intervention in which pharmacists determined their patients at high-risk for overdose based on their prescribed medications and offered counseling on overdose risk and naloxone as well as a passive naloxone-focused intervention that consisted of marketing materials encouraging patients to ask their pharmacist about naloxone. These 2 interventions, and the implementation strategies developed to support their delivery, were pilot tested in rural community pharmacies on relevant implementation outcomes. Given the dearth of research on this topic in rural community pharmacies, we chose to focus the study on rural locations.

This pilot study was made possible by a 1-year pilot award from the UAMS Translational Research Institute, with funds provided by the National Center for Advancing Translational Sciences of the National Institutes of Health (UL1 TR003107). Given the timeline and funding limits of the pilot award, we sought to conduct a small-scale and pragmatic study in partnership with one local pharmacy organization. Our highly partnered approach, characterized by shared decision-making on study elements and co-creation of the interventions and implementation strategies, was consistent with recommended participatory approaches in implementation science.^23^^,^^24^ Our goals for the pilot study were to explore the feasibility, acceptability, and appropriateness of the interventions and supporting implementation strategies as well as their potential to increase the distribution of naloxone among patients at increased risk for overdose.

## Methods

The interventions, implementation strategies, and the overall pilot study approach were developed in a collaborative partnership with Harps, a regional supermarket pharmacy chain with 40 pharmacy locations, 34 of which are located in Arkansas. Harps shares attributes of the independent pharmacy practice setting in that they have smaller locations, smaller prescription volumes, and more personal relationships with patients than larger chain pharmacies.

### Site selection

As part of their collaboration with the research team, the Harps pharmacy district manager was tasked with selecting 2 rural pharmacies to participate in the pilot study and 2 additional pharmacies within their organization that had similar characteristics to the pilot sites (e.g., similar rurality, prescription volume, staffing, and baseline rates of naloxone prescribing/ dispensing). The purpose of the selection of 2 additional non-intervention pharmacies was to serve as comparison pharmacies for external factors that may have influenced dispensing of naloxone (e.g., public service campaigns, changes in opioid prescribing policies, etc.). The 2 intervention pharmacies were selected based on their rural locations and willingness of the lead pharmacists to participate in developing and deploying the interventions and data collection. Prior to the initiation of the interventions, no Harps pharmacy had yet prescribed or dispensed any doses of naloxone. Hence, the baseline level of naloxone distribution at the pharmacies involved in the study was zero.

### Interventions and implementation strategies

Two interventions were pilot tested in the 2 intervention pharmacies: 1) a **proactive opioid overdose counseling intervention** and 2) a **passive naloxone intervention**. Each intervention consisted of adapted and newly created materials and associated implementation strategies detailed below.

The **proactive opioid overdose counseling intervention** was designed to target high-risk patients and initiate conversations about opioids and naloxone. The first step of the intervention began with the pharmacists identifying and “flagging” patients at risk for overdose based on their prescribed medications. Guided by the CDC's guidelines for prescribing opioid for chronic pain, patients were considered eligible if they were: 1) prescribed ≥50 morphine milligram equivalents (MME) per day, or 2) prescribed an opioid medication and 1 or more benzodiazepines, muscle relaxers, or hypnotics for concurrent use.^12^ The pharmacist-in-charge at each intervention pharmacy was trained to query their dispensing software for patients that met these criteria and create alerts that prompted them to invite these patients to receive the opioid overdose counseling intervention when they next visited the pharmacy. A counseling guide with conversation starters and other prompts was developed for pharmacists to reference when delivering the intervention. The counseling guide is provided in Appendix A. When a patient agreed to the intervention, the pharmacist would walk them through an “overdose risk factors” pamphlet and, with help from the counseling guide when needed, explain why they were at risk for overdose. The pharmacist would use a “fire extinguisher analogy” that compared naloxone to a fire extinguisher, explaining that you hope you never have to use it but you have it in your home “just in case” and then recommend naloxone.^25^ If the patient expressed interest in obtaining naloxone, the pharmacist would run the patient's insurance and inform them of the cost. Generally, brand Narcan was recommended by the pharmacist for its ease of use. However, if a patient's insurance would not cover the medication or the patient stated that the cost was too high, the pharmacist would run generic naloxone with the patient's insurance and inform them of the cost. If the patient agreed to purchase naloxone, the pharmacist walked the patient through a second “how to use naloxone” pamphlet and encouraged the patient to train family members how to use it and where it will be kept in the home (using the counseling guide, if needed). When generic naloxone was dispensed, the pharmacist used a naloxone training kit to demonstrate how to assemble the vial and atomizer. When brand Narcan was dispensed, the pharmacist explained that no assembly was required and highlighted links to demonstration videos provided in the pamphlet.

The **passive naloxone intervention** consisted of patient-facing marketing materials to promote awareness and stimulate individuals to ask their pharmacist about naloxone. Materials were adapted from the Maximizing OpiOid Safety with Naloxone (MOON) study^22^ or newly-created to increase patient interest. Specifically, posters were used to target 4 different segments of the population: 1) parents of young children who may accidentally ingest opioid medications in the home, 2) parents of teenagers who may be misusing opioid medications, 3) young couples that may be misusing opioid medications, and 4) elderly individuals and individuals with elderly family members that manage their own medications who may accidentally take more of their prescribed opioids than intended. A large poster was hung on the wall in the waiting area of the pharmacy and a smaller poster was placed on the counter near the register. The large poster being displayed was never the same as the small poster and the posters were rotated every other week. Additionally, a warning sticker was placed on the vial cap of every opioid prescription dispensed, regardless of the MME per day. This warning sticker alerted the patient receiving the medication to the risk for overdose associated with the medication and encouraged them to ask their pharmacist about naloxone.

### Pilot test and implementation outcomes

Guided by Proctor and colleagues' taxonomy of implementation outcomes^26^ and selected collaboratively with our partners, an explanatory sequential mixed-methods design^27^ was utilized to evaluate **adoption**, **feasibility**, **acceptability**, and **appropriateness** outcomes. Our primary adoption outcome measure, collected across both intervention and comparison pharmacies, was number of times *any* naloxone was dispensed by the pharmacies during a 3-month observation period, whether pharmacist- or physician-prescribed (adoption of naloxone). Our pharmacy partners indicated that they considered this measure the most meaningful indication of success of the interventions and the one upon which a future policy decision to “implement system-wide or not” would be made. This measure was determined by querying naloxone dispensing data for each pharmacy and was conducted centrally by the pharmacy partner organization. Data was provided in aggregate as a monthly total of naloxone prescriptions dispensed by each pharmacy.

Within the intervention pharmacies, we specified two additional adoption measures. First, an adoption measure was specified concerning the proactive opioid overdose counseling intervention—the number of patients who received the opioid overdose counseling intervention out of the total number of (at risk) patients approached by the pharmacist. This measure is expressed as the rate of counseling delivered to eligible patients who were approached. Data for this measure were collected by intervention site pharmacists using a spreadsheet provided by the study team. The pharmacists were trained to document 1) their eligible high-risk patients, 2) the date they approached each patient and offered the counseling intervention, 3) whether or not the patient agreed to the intervention, and for those who declined, 4) the main reason for declination (if provided by the patient). The pharmacists provided the de-identified spreadsheet to the study team to determine each pharmacy's number of eligible patients, the number approached, number accepting the counseling intervention, number declined, and a list of reasons provided for declination. Based on these data and the dispensing data described above, we created an additional adoption measure—number of patients who received naloxone out of the number who received the opioid overdose counseling intervention. This measure is expressed as the rate of patients who received naloxone after receiving the counseling intervention. Our pharmacy partner considered this measure to indicate the relative “success” of the proactive opioid overdose counseling intervention.

Intervention pharmacists also documented any individuals not specifically approached by the pharmacist who asked for information about or requested naloxone. When this occurred, the pharmacists were trained to provide the opioid overdose counseling intervention and document whether the individual received naloxone. These data provided information about any naloxone dispensed either as a result of the passive naloxone intervention materials used at the intervention pharmacies (posters and vial cap stickers) or perhaps not as a result of any intervention (i.e, “no intervention/practice as usual,” similar to the comparison pharmacy sites).

To assess feasibility, acceptability, and appropriateness outcomes, semi-structured interviews were conducted 3 months post-implementation with 2 pharmacy staff at each intervention pharmacy. To assess feasibility of the opioid overdose counseling intervention, the interviewer asked questions such as, “How does finding and flagging eligible patients to receive the opioid overdose counseling intervention fit within your workflow?” and “How does prescribing, dispensing, and training patients on use of naloxone fit within your workflow?” To assess acceptability, pharmacy staff were asked, “How complex would you say this intervention was to offer compared to other services you offer?” They were also asked, “How comfortable are you approaching patients about opioid overdose counseling?” and, “What was the interaction like when approaching the patient and offering the counseling?” To assess appropriateness, pharmacy staff were asked whether they felt the pharmacy setting was compatible for this kind of counseling and whether they felt it was a good fit with the role of pharmacists. For example, pharmacy staff were asked, “To what extent were patients welcoming of this kind of intervention and recommendation in the pharmacy?” and, “Do you like this kind of interaction with patients? How well does it fit with your skillset as a pharmacist?” Additionally, general feedback on the intervention materials and implementation strategies was also elicited during interviews. For example, pharmacy staff were asked about what was going well, what barriers they faced, whether they needed additional materials or support, whether training was adequate or should be improved, what kind of feedback they would like to determine how well they were doing, and what recommendations they had for improvement. The full interview guide is provided in Appendix A. A rapid template analysis technique based on methods described by Sobo and colleagues^28^ was utilized. This technique is commonly used in health services and implementation research to explain implementation phenomena. Two research team members utilized an interview summary template that allowed for coding interview responses into categories based on implementation outcomes of interest selected a priori while providing the opportunity for emergent themes as well.

## Results

Between the 2 intervention pharmacies, a total of 148 unique patients were offered the opioid overdose counseling intervention. Of those offered the intervention, 130 agreed (87.8%) to the opioid overdose counseling intervention (Table 1). A majority (83.8%) of the patients that received the opioid overdose counseling intervention were prescribed an opioid in combination with 1 or more benzodiazepines, muscle relaxers, or hypnotics. Only 2 of the individuals who received the opioid overdose counseling intervention were not considered high-risk according to CDC guidelines but asked their pharmacist about naloxone as a result of the warning sticker on the cap of their opioid prescription.

**Table 1:** Patient Conversations about Naloxone and Purchasing Decisions.

Total			n (%)
	Talked with Pharmacist		130/148 (87.8)
		Purchased Naloxone	44/130 (33.8)
By Pharmacy			
	Talked with Pharmacist		73/82 (89.0)
		Purchased Naloxone	25/73 (34.2)
	Talked with Pharmacist		57/66 (86.4)
		Purchased Naloxone	19/57 (33.3)

Overall, 44 (33.8%) patients that received the opioid overdose counseling intervention were prescribed and dispensed naloxone. Of the patients prescribed an opioid medication ≥50 MME per day, 3 out of 19 (15.8%) were dispensed naloxone (Table 2). Patients coprescribed an opioid and 1 or more benzodiazepines, muscles relaxers, or hypnotics, were dispensed naloxone at a higher rate (36.7%; 40/109). During the study period, 0 naloxone prescriptions were written or dispensed at the comparison pharmacy sites.

**Table 2:** Patients Purchasing Naloxone by Medication Regimen.

Medication Regimen	Purchased Naloxone n (%)
Over 50 MME per day ONLY	2/11 (18.2)
Over 90 MME per day ONLY	1/8 (12.5)
Under 50 MME + Other Drug	29/69 (42.0)
Over 50 MME + Other Drug	10/32 (31.3)
Over 90 MME + Other Drug	1/8 (12.5)
Not High-Risk	1/2 (50.0)

**Total**	**44/130 (33.8)**

Qualitative interviews with 4 pilot pharmacy staff took place 3 months post-implementation. The pharmacy manager from each pharmacy, 1 staff pharmacist, and 1 pharmacy technician participated. In regards to feasibility, all participants reported that the both interventions fit well within their normal workflow after taking some time to adjust. For example, 1 staff member stated, “during the first 4 to 6 weeks, that's really when… you know, we had multiple people that we had to ask about naloxone every day. But after that, we knew who was interested so we could follow up with them… and there was less than a handful of new patients that we'd need to ask each month.” A suggestion to reduce the initial heavy workload was to focus on one population (e.g., only those with opioid/benzodiazepine or opioid/muscle relaxer) when starting the intervention.

Regarding acceptability, pharmacy staff made a few suggestions that they felt would improve patients' feelings about the intervention but thought patients were willing to talk and accept the recommendation to purchase naloxone if the conversation wasn't accusatory. A staff member explained, “…making the conversation about family… like, children or grandchildren might get into your medicine… and not pointing the finger at the patient…That's when we had better success.” Additionally, introducing the fire extinguisher analogy to explain naloxone early in the conversation was seen as a helpful way to help patients understand why they were being asked to talk with the pharmacist about their opioid medications.

As appropriateness was concerned, all staff members thought approaching patients to provide education about opioid medications and overdose risk in the pharmacy was an appropriate setting. However, one pharmacist described a conversation with a local physician that was unpleasant. They explained, “he called up here and was all, ‘why are you telling my patient they need naloxone?’ and he was saying I was scaring his patient… but I just told him, listen, I'm doing it because the medications you prescribed put them at risk.” When asked what could be done to avoid similar situations in the future, it was suggested that a letter be sent to physicians in the area when starting the program to inform them in advance of this pharmacy-based initiative.

Pharmacists recorded the reasons patients refused the naloxone prescription on the computerized spreadsheet. The most frequently reported reasons for not dispensing naloxone were cost/insurance-related (e.g., copay was too high, required prior authorization, other insurance issues), patient had low perceived risk of overdose (e.g., taking the same medications for years, kept medications in a locked medicine cabinet or safe, medications managed by another person), and patient wanted to bring the pamphlet home to read more and/or discuss with family. Pharmacy staff felt cost was a major barrier for patients with 1 interviewee stating, “if it was under $10.00, I bet at least another third of my patients would have left with naloxone.”

## Discussion

This small-scale pilot study of 2 interventions to reduce overdose risk from opioids in community pharmacies yielded largely positive results. The proactive intervention resulted in pharmacists providing the opioid overdose counseling intervention to 130 patients over 3 months, and among those patients, 44 naloxone prescriptions were written and dispensed. In terms of the passive intervention, however, only 2 individuals responded to the posters and/or vial cap stickers and requested more information about naloxone. While passive materials were found to be beneficial in previous research,^22^ it is possible that a more proactive approach is more important for pharmacies located in the rural US with patients taking a large number of prescribed opioids (and other contra-indicated medications) but fewer illicit drug users presenting.^29^ However, the posters and vial cap stickers may have created awareness of the dangers of opioids and, although they did not result in a large number of conversations about naloxone, may have been seen as beneficial to patients. Additionally, data was not collected on the number of opioid prescriptions that received a warning sticker that were not considered high risk. Therefore, it is possible that individuals who received the proactive opioid counseling intervention may have responded to the sticker had the pharmacist not approached them first. A future study that utilizes appropriate designs (e.g., cluster randomized trial, stepped wedge) to tease out individual intervention pieces and test a “basic/passive” intervention (e.g., posters and stickers only) to a “basic + enhanced/proactive” intervention (e.g., posters and stickers, + the proactive approach) would be beneficial to determine where resources should be allocated to achieve the best results and dispense the most naloxone.

Many studies discuss lack of time as a barrier to providing interventions in the community pharmacy setting due to the already high demands of regular dispensing activities.^30^^,^^31^ However, pharmacy staff in this study reported the majority of the work was done within the first 4–6 weeks after implementation. After the initial “surge” of eligible patients who were identified and approached, interventions were only provided to the few new patients each month meeting the high-risk criteria. It is important to highlight this finding when attempting to disseminate similar interventions to community pharmacies or when planning a larger implementation trial of these interventions. While some may view the proactive opioid overdose counseling intervention as overly time consuming initially (especially in community pharmacies with higher daily prescription volumes than our regional supermarket pharmacy chain partner), if the pharmacy staff can plan accordingly to work through the relatively high number of existing at-risk patients at “baseline,” the practice can more easily become routinized with a relatively low number of newly-occurring at-risk patients.

While the research team and pharmacy partner found the 34% naloxone acceptance rate among patients receiving the opioid overdose counseling intervention to be positive and promising for future exploration of these interventions, it is important to note that a majority of high-risk patients who received the opioid counseling intervention *were not* dispensed naloxone. Clearly, there is room for improvement with these interventions and their supportive implementation strategies. Additionally, qualitative interviews with pharmacy staff found that cost likely contributed to many patients refusing naloxone. A number of states have mandated naloxone be coprescribed for patients on high-risk opioids and patients prescribed concomitant opioids and benzodiazepines. In these states, research has found an increasing number of payors have placed naloxone on their formularies leading to low-cost or no-cost naloxone.^32^ This study adds to the literature supporting such mandates to ensure patients who need naloxone can obtain it at a reasonable cost.

This study has several limitations. First, this study was conducted in only 2 rural community pharmacies with 2 comparison pharmacies that were members of the same regional supermarket chain in the southern US. This was a highly collaborative, pragmatic pilot study and therefore our pharmacy partner was highly involved in the development and implementation of the interventions. As stated in the methods, the 2 intervention pharmacies were purposefully selected by the pharmacy district manager because of the willingness of the pharmacist-in-charge to help with the development of the interventions and data collection. Therefore, findings from this small pilot study may suggest some positive potential of these interventions but are not generalizable. Pharmacies located in different regions or practicing in other pharmacy types may not achieve similar results or observe similar themes. Additionally, we did not collect data on length of employment from pharmacy staff at the intervention or comparison pharmacies. It is possible an established (or lack of) pharmacist-patient relationship could impact the results of an opioid counseling intervention. Future research utilizing more rigorous study designs is needed to better understand barriers to proactive opioid overdose counseling interventions in the US. Second, as mentioned above, data were not collected that could be used to determine whether the passive naloxone intervention would have resulted in naloxone being prescribed and dispensed had the pharmacist not proactively intervened. Third, because of the low number of intervention sites, qualitative interviews were only conducted with 4 pharmacy staff involved in providing the intervention. Themes are likely specific to these 2 pharmacies. However, we hypothesize that cost is a barrier in states that do not have a naloxone coprescription mandate. Fourth, only 3 months of data post-implementation of the interventions were collected and analyzed. Pharmacists reported that they thought they had approached all patients meeting the high-risk criteria in this time and additional interventions would only occur with new patients. However, sustainability of the interventions and implementation of these interventions to multiple pharmacy types and settings should be explored in a larger randomized controlled trial.

## Conclusion

This highly-partnered pilot study of a proactive opioid overdose counseling intervention and passive naloxone intervention in 2 intervention pharmacies was positive with a large majority of at-risk patients (87.8%) agreeing to receive an opioid overdose counseling intervention and a third of them (33.8%) were dispensed naloxone following a pharmacist's recommendation. The majority of patients who received the opioid overdose counseling intervention did not receive naloxone indicating additional revisions to the intervention components and/or implementation strategies are needed to improve the overall impact of the interventions. The participating pharmacy staff found the interventions to be feasible, acceptable, and appropriate for their settings and workflows. Interventions in rural community pharmacies can increase the reach of naloxone by identifying and proactively approaching high-risk patients and are greatly needed to help prevent deaths caused by opioid overdose.

## Funding

This study was funded by the 10.13039/100015317UAMS Translational Research Institute (TRI), grant U54TR001629, through the 10.13039/100006108National Center for Advancing Translational Sciences (NCATS) of the 10.13039/100000002National Institutes of Health (NIH). The content is solely the responsibility of the authors and does not necessarily represent the official views of the NIH.

## Declaration of Competing Interest

All authors declare no conflicts of interest or financial interests in any product of service mentioned in this article, including grants, employment, gifts, stock holdings or honoraria.

## Appendix A Supplementary data

Pharmacy Staff Interview Guide Image 1

Counseling Guide Image 2
