# Supplementary material for: Opioid overdose counseling and prescribing of naloxone in rural community pharmacies: A pilot study
Source: Explor Res Clin Soc Pharm. 2021 May 3;2:100019. doi: 10.1016/j.rcsop.2021.100019 (PMC8813166; doi:10.1016/j.rcsop.2021.100019)
Supplement: Supplementary file 1 — Pharmacy Staff Interview Guide [file mmc1.docx]

| ***Pharmacy Staff Interview Guide*** |
| --- |

- What barriers are there to prescribing and dispensing naloxone in your pharmacy?
- How does prescribing, dispensing, and training patients on use of naloxone fit within your workflow?
- How was the demand for the naloxone after implementing the materials and strategies?
  - Did you think the posters advertising naloxone in the pharmacy were useful?
    - Why or why not?
    - What would you do to improve them?
  - Did you think the sticker on the bag was useful?
    - Why or why not?
    - What would make it better?
- How does finding and flagging eligible patients to receive the opioid overdose counseling intervention fit within your workflow?
  - How would you improve the identification of potential patients that might benefit from naloxone?
- How complex would you say these interventions were to offer compared to other services you offer?
- What other materials do you think may help get patients to ask about naloxone while in the pharmacy?
- What would you change to improve the interventions?
- How comfortable are you approaching patients about opioid overdose counseling?
- What was the interaction like when approaching the patient and offering the counseling?
- To what extent were patients welcoming of this kind of intervention and recommendation in the pharmacy?
  - Was there any negative feedback from them?
  - What were the reactions of the patients?
  - How would you improve the interaction?
  - For those that rejected your recommendation, what do you feel the most common reason was?
- How did you counsel the patients about the naloxone?
- Do you like this kind of interaction with patients? How well does it fit with your skillset as a pharmacist?
- Did you demonstrate how to prepare and use the naloxone?
- Did patients typically pay for the naloxone? How much?
- If you have any questions about how to order, prescribe, dispense, or counsel about naloxone, who would you contact?
- What would you need to be able to continue prescribing and dispensing naloxone?
