# Supplementary material for: Opioid overdose counseling and prescribing of naloxone in rural community pharmacies: A pilot study
Source: Explor Res Clin Soc Pharm. 2021 May 3;2:100019. doi: 10.1016/j.rcsop.2021.100019 (PMC8813166; doi:10.1016/j.rcsop.2021.100019)
Supplement: Supplementary file 2 — Counseling Guide [file mmc2.pdf]

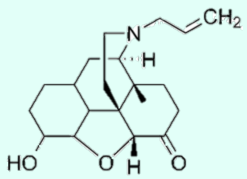

A GUIDE FOR PHARMACISTS DISPENSING NALOXONE TO PATIENTS

❖ Easy to use instructions for dispensing naloxone to patients.

1. CONVERSATION STARTERS

➢ If it's alright with you, I'd like to talk to you a little bit about your opioid prescription and the possibility of getting some naloxone to take home with you as well.

FOR THE AT-RISK PATIENT

- Opioids can cause bad reactions that make your breathing slow or even stop. This can happen if your body can't handle the opioids you take that day or if you take opioids with alcohol or other drugs. Naloxone is a lifesaver just like a seatbelt or a fire extinguisher. Everyone needs to have it if they are at risk.

FOR OLDER PATIENTS THAT MAY BE FORGETFUL AND ARE AT RISK FOR ACCIDENTAL OVERDOSE

- These medications can be helpful but have a range of side effects, like slowing down or even stopping breathing completely. Naloxone can help if this happens by restoring breathing.

FOR THE PATIENT WITH CHILDREN/ GRANDCHILDREN

- Opioid medications increase the risk of breathing emergency with a person who takes the opioid and anyone in their household. Naloxone is needed in case of emergency. Let's keep you and your family as healthy as possible with these medications in your house. Just in case, get naloxone.

2. WALKING THE PATIENT THROUGH THE PAMPHLET

An overdose from opioids causes a person to breath slower or even stop breathing and it is very difficult to wake a person from this state. This can take minutes to hours to develop which means there is time to respond and help someone.

- There are a number of different factors that can put you at increased risk for overdose.
- For example, it looks like you________
    I. Are prescribed a high dose of opioids
    II. Have been on opioids for a long time
    III. Have a prescription for an opioid and a benzo/muscle relaxant/sleeping pill
    IV. Have a history of COPD or other reparatory illness
- And this will put you at greater risk. Also, having these medications in your home puts any children or relatives at risk if they accidentally ingest some of your medication.

THINK OF IT LIKE A FIRE EXTINGUISHER – YOU HOPE YOU NEVER HAVE TO USE IT BUT YOU HAVE IT IN YOUR HOME JUST IN CASE.

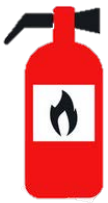

3. IF PATIENT REQUESTS A NALOXONE PRESCRIPTION, TURN OVER FOR DISPENSING/COUNSELING TIPS

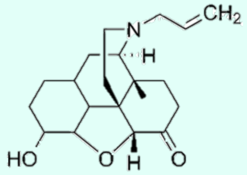

A GUIDE FOR PHARMACISTS DISPENSING NALOXONE TO PATIENTS

- Easy to use instructions for dispensing naloxone to patients.

COUNSEL ON HOW TO RESPOND IN AN OVERDOSE AND HOW TO USE NALOXONE

If you suspect someone is having an opioid overdose, first check for the signs listed here. Try to see if you can wake them up by yelling and shaking them to see if they respond. If you aren’t 100% sure that they are having an overdose, you should treat the situation as if they are having an overdose. Immediately call 911 and then administer the naloxone. It is VITAL that you call 911 because naloxone is a TEMPORARY fix that will wear off quickly. That means the opioids will still be in the person’s system and they will go back into overdose without further medical treatment to keep them stabilized.

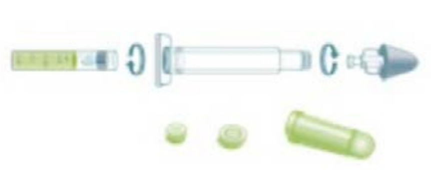	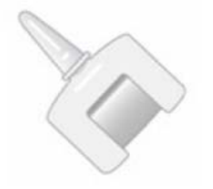
**Multi-Step Nasal Spray**	**Single-Step Nasal Spray (Narcan)**
**Quantity: 2 doses**	**Quantity: 2 doses**
**Dispense two (2) 2 mL prefilled needleless syringe plus two (2) mucosal atomizer devices (MAD-300).**	**Dispense one (1) two-pack of two (2) 4mg/0.1 mL intranasal devices.**

To administer the Naloxone with Atomizer:

Demonstrate how to assemble the naloxone and atomizer. Once that is shown, be clear that half of the vial is to be sprayed into each nostril.

To administer the NARCAN Nasal Spray:

Explain that there is no assembly required and all they need to do is insert the tip into one of the individual’s nostrils and push the plunger on the bottom.

- Make sure you stay with the person and watch to see if they begin to come out of the overdose. If they are still unresponsive after 3 minutes, you should give them a 2nd dose of naloxone. Continue to stay with the person until help arrives. When possible, turn the person on their side. This lessens the chance that the person will choke on vomit if they begin to come out of the overdose. Make sure not to leave the person until help has arrived. The law gives immunity protection for individuals who are administering naloxone to a person suspected to be in an opioid overdose so there is no risk to you for doing this and trying to save someone’s life.
- There are multiple videos available at the links listed here in the pamphlet. Please watch the appropriate videos for the type of naloxone we are giving you. Also, share this information with others in your household and make sure that everyone knows where the naloxone is located and how to use it. I’d recommend having the other members of your household watch these videos as well to make sure that if you have an accidental overdose, they will know how to respond.
